# Barriers and Facilitators of Using MyDispense from the Student Perspective: A Systematic Review

**DOI:** 10.3390/pharmacy13060158

**Published:** 2025-11-01

**Authors:** Owen Collins, Ruth McCarthy, Laura J. Sahm

**Affiliations:** 1 Pharmaceutical Care Research Group, School of Pharmacy, University College Cork, T12 K8AF Cork, Irelandruth.mccarthy@ucc.ie (R.M.); 2 Pharmacy Department, Mercy University Hospital, Grenville Place, T12 WE28 Cork, Ireland

**Keywords:** MyDispense, computer-based simulation, pharmacy education, pharmacy students

## Abstract

MyDispense is a high-fidelity, low-stakes community pharmacy simulation, allowing students to practice dispensing skills. A systematic review was conducted to identify students’ perceptions regarding barriers and facilitators of MyDispense in pharmacy education. PubMed, CINAHL, and EMBASE databases were searched from 2015 to 2025 in January 2025 using combined keywords, proximity searching and Boolean operators. Studies investigating MyDispense and gathering students’ perceptions were included. Record screening was conducted by two independent reviewers (OC and LS). Any identified records from database searching and hand searching of included study reference lists were imported to Rayyan and subjected to independent review. Conflicts were resolved through a third party (RMcC), and discussions were held until consensus was reached. Fifteen studies were included in this review. Seven studies were conducted in USA, six in Asia, one in UK, and one in Australia. All studies utilised purposive sampling. Sample sizes ranged from 33 to 322 students. All studies included surveys to gather student perceptions. Other data collection methods included semi-structured interviews and focus group discussions for students to further elaborate on survey responses. Identified facilitators were mapped to four overarching themes; “Develops competency”, “User-Friendliness”, “Engaging Learning Experience” and “Safe Learning Environment.” Key barriers were encompassed to three themes: “Learning Curve”, “IT issues” and “Limited Realism and Applications”. Barriers included (i) the learning curve of the platform, (ii) technical issues, and (iii) limited realism. Facilitators included perceptions of (i) improved dispensing and counselling skills and a deeper understanding of pharmacy legislation, (ii) accessibility, interactivity of the learning environment and (iii) immediate feedback. Synthesis of the evidence in this review identified students’ perceptions of barriers and facilitators of MyDispense in pharmacy education. This may serve as a guide to educators considering the adoption of MyDispense into their curricula.

## 1. Introduction

Pharmacy students need to complete a curriculum aligned with accreditation standards to ensure they demonstrate the competencies required as a pharmacist [1,2]. Pharmacy practice can be simulated through a wide range of pedagogical methods, e.g., workshops, experiential learning opportunities and using standardised patients in observed structured clinical examinations (OSCEs). Logistical challenges associated with incorporating actors as patients to simulate clinical practice, however, may limit their applications and novel approaches are required to meet pharmacy curricular outcomes [3].

Simulation has been described as a method rather than a technology and was developed to replicate or enhance real-life experiences through guided, sometimes immersive, scenarios that aim to mirror key aspects of the real world in an interactive way. In this context, simulation involves using tools such as standardised patients or mannequins to train healthcare professionals when learning specific skills or competencies [4]. A comprehensive overview of the implementation and learning outcomes of simulation in pharmacy education, in 2023, concluded that simulation has substantial potential to further advance practice-based learning outcomes across diverse cohorts [5].

Simulation-Based Training (SBT) offers learners the chance to engage in practice in a controlled, risk-free environment. It enables them to respond to high-stakes situations, make mistakes, and learn from those errors without compromising patient safety. By using simulation, educators can avoid placing real patients in repeated training scenarios or exposing them to potential harm from trainee errors [6,7]. Before 2000, pharmacy education relied heavily on lectures and textbooks. Simulation was minimal, using physical models or role-play. In the early 2000s, possibly inspired by aviation and medical training, pharmacies began adopting SBT to enhance clinical decision-making and patient safety. Applications of SBT for pharmacists can vary from cannula insertion, blood pressure monitoring, or prescription review to high-impact multidisciplinary team simulation for the management of clinical scenarios [8].

Technological advancements have enabled pharmacy students to engage in patient care simulations without needing to be physically present in a pharmacy setting. Computer-Based Simulation (CBS) tools and applications bring these experiences into the classroom in a dynamic and interactive way [9,10]. CBS can be defined as an interactive computer simulation model of patient–clinician interactions that allows learners to emulate healthcare professional roles to obtain a full patient history, conduct physical health check-ups and to make appropriate diagnostic and therapeutic decisions [11]. Active learning methods, e.g., CBS, can be more effective at improving student performance in formal assessments relative to passive learning methods [12]. Alternative learning methods are particularly important for Generation Z (those born between 1997 and 2012 [13]) who are currently the primary demographic cohort of students within third-level education. This digitally literate generation tends to benefit from blended learning approaches [14].

Virtual Reality (VR) is a form of CBS that uses simulation software to help students practice specific tasks or navigate complex scenarios [15,16]. These tools integrate multimedia elements such as audio, visuals, animation, and text to create immersive learning environments. VR simulations can replicate real-world settings like clinical environments, community pharmacies, or interactions with virtual patients. MyDispense is an example of such a programme. [7,17] MyDispense and other CBS tools experienced greater implementation during the COVID-19 pandemic, as they provided engaging learning experiences whilst enhancing digital literacy. [18,19,20,21].

MyDispense is a high-fidelity, low-stakes, web-based community pharmacy simulation developed by the Faculty of Pharmacy and Pharmaceutical Sciences at Monash University in Victoria, Australia [22]. We have focused on MyDispense for many reasons, including the evidence that it (i) has a global reach, being implemented in over 200 institutions across 30 countries worldwide [22], (ii) allows students to develop their dispensing and counselling skills without the risk of patient harm in a virtual pharmacy sandbox environment [23,24] (iii) can be used in senior years of pharmacy programmes as a supplementary learning resource to prepare students for OSCEs and pre-registration assessments and (iv) allows for the sharing of practices, ideas and materials within the MyDispense community.

Virtual patients act as a novel nexus between clinical theory and practical applications for students, acting as an accessible alternative to standardised patient actors and experiential learning opportunities, whereby recruitment and scarce placements can pose logistical challenges [25]. MyDispense supports active self-learning by providing instant feedback and repeat exercises, reinforcing critical thinking, clinical reasoning and problem solving skills [26]. These skills align with Kolb’s learning model and Miller’s learning framework, meeting pharmacy programme outcomes [26]. Despite these advantages, the use of virtual patients in pharmacy education appears to be relatively low, possibly due to implementation barriers [27].

Previous reviews conducted on pharmacy CBS programmes have varied in their focus. Virtual Patient Simulation (VPS) in pharmacy education, and the effect of adapting VPS, on pharmacy students’ engagement, confidence, knowledge, skills, and satisfaction was examined by Beshir et al. [28]. The different computer simulators and their respective designs, available in CBS in pharmacy practice education, were the subject of a narrative review, which concluded that educators should reflect on their specific institutional, professional and curriculum needs before choosing the product, so that it aligns with their teaching goals [25].

Appraisal of the current applications of MyDispense within pharmacy education to inform practice, use and future development was the subject of the review conducted by Khera et al. [29]. They concluded that MyDispense was used to enhance student learning, increase academic and practical knowledge, develop essential skills needed to become a pharmacist and to support educators in their teaching. Our review builds upon this as it addresses a gap in the literature by exploring the student perspective on barriers and facilitators of MyDispense in pharmacy education. Therefore, to guide future research and implementation strategies for educators, the aim of this systematic review is to synthesise relevant literature to identify students’ views regarding the barriers and facilitators of MyDispense in pharmacy education.

## 2. Materials and Methods

### 2.1. Search Strategy

The Preferred Reporting Items for Systematic Reviews and Meta-Analyses (PRISMA) guidelines were used to conduct this systematic review (Appendix A) [30]. The PICO (P: Population; I: Intervention; C: Comparisons; O: Outcomes;) framework was applied to further define our research question and identify relevant search terms (Table 1).

A systematic search of PubMed, CINAHL and EMBASE from 2015 to 2025 was performed in January 2025 using combined keywords, indexing terms and proximity searching. Boolean operators (AND, OR) were used to refine the search, as were Medical Subject Headings (MeSH) terms in PubMed and CINAHL plus Emtree terms in Embase. This date range was chosen as it encompasses the most recent ten years of research. Common search terms used across all three databases in this systematic review were “MyDispense”, “computer simulation”, “patient simulation”, “pharmacy students”, “perceptions”, “facilitators”, “enablers”, “barriers”, and “pharmacy education”. Examples of the search strategy used across all the databases can be found in Appendix B. Manual hand-searching of included studies reference lists, identified from database searching was performed to seek out any further additional relevant studies to be included for review.

### 2.2. Eligibility Criteria

Studies were included if they met the following criteria for inclusion:

1. Primary research sources;
2. Published between January 2015 and January 2025;
3. Qualitative, quantitative and/or mixed-methods studies examining pharmacy students’ perceptions of MyDispense;
4. Published in English.

Studies were excluded if they met the following criteria for exclusion:

5. Reviews, conference abstracts, meta-analyses, commentary studies, grey literature;
6. Not published in English;
7. Not investigating the use of MyDispense;
8. Did not include a pharmacy student population.

### 2.3. Study Selection

References from all three databases were imported into Rayyan [31]. Any duplicate studies were removed. Title/abstract screening was conducted by two independent reviewers (OC and LS) against the pre-determined inclusion and exclusion criteria. Thereafter, full text studies were retrieved for screening by two independent reviewers (OC and LS) for inclusion. Any identified studies from reference list searching were imported to Rayyan and subject to full-text screening by the reviewers (OC and LS) for inclusion. Any conflicts that arose were resolved through a third party (RMcC), with discussions being held until a consensus was reached.

### 2.4. Data Extraction and Synthesis

Thematic analysis by the specific approach outlined by Braun and Clarke was performed to identify barrier and facilitator themes to provide further insight on student perceptions [32]. Full texts were imported into NVivo 15.1.1 to facilitate thematic analysis. Data extraction was conducted by OC on all included studies. This was cross-checked, on a specified sample of 20%, by LS for accuracy. Qualitative and quantitative results (with a degree of qualitative insight) from included studies were coded to identify possible barrier/facilitator themes. All study characteristics (author(s), year of publication; jurisdiction; study design; outcomes; participants; data collection methods; facilitators; barriers) were collected.

### 2.5. Quality Assessment of Included Studies

The methodological quality of all included quantitative, qualitative and mixed-methods studies were critically appraised by two authors independently (O.C. and L.S.), according to the Mixed Methods Quality Appraisal Tool (MMAT) quality criteria [33]. Consensus on the MMAT was achieved through a collaborative and iterative process involving both authors. We began by reviewing the tool’s criteria and discussing its applicability to our specific context. Both authors contributed insights based on their expertise and methodological perspectives. Through a series of structured discussions, we identified areas of agreement and clarified points of divergence. Where conflicts arose, a consensus was reached through discussion between both reviewers. No studies were excluded, regardless of their quality appraisal outcome. 

## 3. Results

### 3.1. Studies Eligible for Inclusion

**Figure 1:**
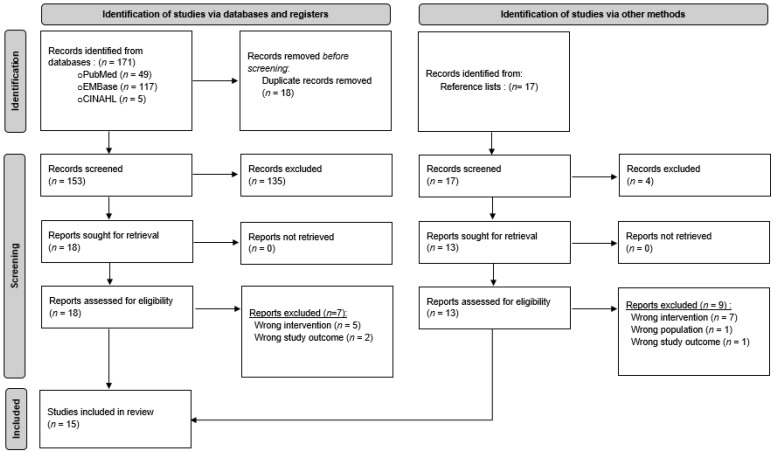
PRISMA flow chart.

Initial database searches yielded 153 records, following duplication removal. In total, 18 studies met the eligibility criteria and were included for full-text screening. Following independent review, 7 of the 18 full texts were excluded. A total of 17 studies were identified from manual hand-searching, including full-text citation lists, and 4 studies were included in the review. A diagram outlining the flow of studies within this review can be seen in Figure 1.

### 3.2. Characteristics of Included Studies

Fifteen studies were included in this review. Seven of the fifteen studies were conducted in USA [34,35,36,37,38,39,40], six in Asia [41,42,43,44,45,46], one in UK [47], and one in Australia [26]. All studies used purposive sampling. Sample sizes ranged from 33 [46] to 322 [44] students. The average number of participants across all studies was 121 students. Ten studies employed a mixed-methods approach [26,34,37,39,41,43,44,45,46,47] and five studies used a quantitative methodology [35,36,38,40,42]. All studies were questionnaire–based using closed-ended, open-ended and Likert-scale questions to gather student perceptions. Other data collection methods included semi-structured interviews [42] and focus group discussions [46]. Seven studies were longitudinal [35,36,38,40,41,42,43] and eight studies were cross-sectional in nature [26,34,37,39,44,45,46,47]. All studies included pharmacy students. One study included pharmacy instructors [41] and another included pharmacists with one year of experience [43] in the study population. An overview of the study characteristics can be found in Table 2.

### 3.3. Summary of Identified Facilitators

The evidence indicated that facilitators identified include improved dispensing and counselling skills and a deeper understanding of pharmacy legislation. MyDispense is an accessible, interactive and engaging learning environment for students. Instant feedback at the end of exercises appeared to promote active learning. Analysis of the included studies revealed that students appreciated the risk-free environment of MyDispense. The outlined facilitators identified in this review were mapped to the four themes: (1) Develops Competency, (2) Accessibility, (3) Engaging Learning Experience, (4) Safe Learning Environment.

#### 3.3.1. Facilitator Theme I: Develops Competency

Across the reviewed literature students reported that MyDispense enabled them to practice skills needed to correctly and safely dispense medications, e.g., appropriate labelling [34,35], verifying patient identities [34,35,36,37,38,39,40,41,42,43], identifying prescription errors and omissions [34,38,39], and referencing appropriate information sources [37,43,44]. This was reported to be of particular help to those without prior community pharmacy experience “*I think this is a neat and useful tool for pharmacy students to learn before their community pharmacy rotation, especially for those who have never had experience in a community pharmacy before*” [39].

The evidence indicated that MyDispense helps students systematically organise their thoughts when dispensing, which likely fosters best practice habits [45]. Most students (97.1%) agreed/strongly agreed that MyDispense helped them better understand the steps required to dispense prescriptions safely [26] and familiarise themselves with products: “*It helps me get used to some brand names, because its less common when I’m studying*” [43].

Analysis of the included studies revealed that MyDispense develops patient communication skills [35,36,41,43,44,47]. A majority (71.1%) of first year students reported increased OTC knowledge and counselling skills upon completing MyDispense exercises [47]. Positive perceptions were also observed in students in the senior years of pharmacy programmes, as 70.1% of fourth and fifth year students felt it was effective for the development of counselling skills [43].

The implementation of MyDispense to support pharmacy law skill development was reported in four studies [34,37,39,47]. The vast majority (86.9%) of students across two years of a PharmD programme agreed that MyDispense helped active recall of pharmacy laws from didactic lectures, and most (73.2%) reported that this application of MyDispense enabled them to enhance their understanding of pharmacy law [37]. Evidence indicate that MyDispense allowed students to become familiar with brand-names of medicines encountered frequently in practice within their jurisdiction [36,43,47], which may provide a smoother transition to practice.

#### 3.3.2. Facilitator Theme II: Accessibility

According to the evidence provided, MyDispense is widely accessible, allowing students to practice exercises in their own time and from any location [35,42,44,46]. Three studies highlighted its remote accessibility and use during the COVID-19 pandemic [41,44,47]. One third of students in one study felt that being able to practice dispensing at any place or time was one of its most useful features [40]. MyDispense can be accessed from mobile devices which further facilitates its remote use by students: “*I liked that MyDispense can be used in my phone so I can do it anywhere when I have time*” [24].

#### 3.3.3. Facilitator Theme III: Engaging Learning Experience

The included studies demonstrated that MyDispense offered a high-fidelity, virtual pharmacy learning environment to support students, particularly for those with no prior pharmacy experience [37,38,39,41,43,44]. Pharmacy students further expressed appreciation for the realism of the simulation experience which may be a useful enabler for learners who benefit from visual aids: “*I could observe patient appearance including their ages, gender and other special features such as pregnant women, so it helps me visualise better*” [43]. Nearly three out of four PharmD students across three US institutions agreed/strongly agreed that MyDispense was more realistic than paper-based cases [39]. Additionally, 84.4% of students in one study reported that MyDispense was a stimulating learning environment [26].

The synthesis of findings showed that students can actively learn from the instant feedback feature of MyDispense [26,34,40,41,43,46]. This increased student confidence as they can use such feedback to change their approach in subsequent exercises and promotes autonomous learning within pharmacy students [26]: “*One function that I find very cool is the feedback, which helps me have the ability to self-study and self-check whether the prescription I give to the patient is incorrect or not*” [43]. Most (83.4%) students expressed agreement that prompt feedback was helpful for improving their understanding [26]. Likewise, 83.6% of students in another survey reported prompt feedback provided by MyDispense as one of its most useful features [42].

#### 3.3.4. Facilitator Theme IV: Safe Learning Environment

Students reported that MyDispense provided a controlled learning environment whereby they can make mistakes [26,36,39,40,41,43,44]. Two studies reported that providing a safe environment to practice was one of the commonly cited facilitators by students, particularly for novice students prone to mistake [40,42]. Students also reported appreciation for the ability to repeat exercises, which can facilitate active learning from mistakes and reinforce learning from exercises [43,44,47]: “MyDispense is good because it gives us the experience and practice of realistic dispensing without having to place any risk on real patients in our community.” [26].

### 3.4. Summary of Identified Barriers

The evidence suggested that barriers included the initial difficulties of navigating MyDispense and some students felt the user interface (UI) could be improved to provide a more interactive experience. Technical issues also caused student frustration. MyDispense only replicates community pharmacy practice and students reported that patient–prescriber interactions were not authentic because oral communication is not a platform feature. The outlined barriers, based upon the synthesis of the findings, were aligned to three themes: (1) Learning Curve, (2) Information Technology (IT) issues, and (3) Limited Realism and Applications.

#### 3.4.1. Barrier Theme I: Learning Curve

Across the reviewed literature, eight studies reported students felt MyDispense ^TM^ was difficult to use initially [34,38,39,41,43,44,45,47]. Students reported and highlighted the need for training on the platform to facilitate its use, suggesting that tutorials may help overcome the initial learning curve of the platform: “*A tutorial version of these cases where you learn as you go instead of after you finish the entire case may be helpful*” [39]. In one study, a third of students (33.8%) reported that more instructions would have been required prior to use. Similarly, only half of all Vietnamese pharmacy students questioned agreed/strongly agreed that MyDispense was straightforward to use [43,47].

Evidence indicated that students reported that the design and appearance of the UI could be improved to provide a more learner-friendly experience [26,43,47]: “*Improvement of the design of the user interface of MyDispense for easier navigation and better appearance of the application for the user*” [44]. Label fonts were reportedly difficult to read and product images were occasionally of poor resolution, negatively impacting simulation fidelity [41,43,44]. The UI was not optimised for Thai and Vietnamese learners, as English was the only available language in MyDispense [41,43].

#### 3.4.2. Barrier Theme II: IT Issues

Analysis of three studies demonstrated that students had limited MyDispense access due to internet connectivity issues [26,41,44]. One study reported a significant relationship between internet connectivity and MyDispense use (*p* < 0.001), whereby an increase in internet connectivity is associated with a higher percentage of student MyDispense participation [44]. Students also reported minor technical issues and gave feedback that MyDispense was incompatible with certain devices and web browsers [26,38,44,46]—“*We had to use a certain web browser and it would become very confusing when trying to back out or submit medication*”—therefore potentially limiting its use or negatively impacting the overall learning experience of the simulation [34].

#### 3.4.3. Barrier Theme III: Limited Realism and Applications

Within the findings of four studies, students reported that MyDispense was limited in its capabilities as it only simulated community pharmacy practice and did not offer the opportunity to be exposed to other areas of clinical practice [40,42,47]: “*A possible improvement is the option to be exposed to different kinds of pharmaceutical workplace settings, like the option to pick between settings like Hospital Pharmacy or Community Pharmacy*” [44]. Two studies reported this feature as one of the least useful design aspects [40,42]. Evidence from the studies also suggested that students wanted more varied exercises, e.g., veterinary prescription exercises, for a more comprehensive and integrated learning experience [47]. Students suggested that MyDispense could be more relevant to practice by including a commercially available dispensing software within the simulation [26,47].

Some studies suggested students felt patient and prescriber interactions within MyDispense were limited in nature [40,42,45,47]. Students reported that interactions did not feel authentic, as oral communication is not a feature of MyDispense [45]. Overall, 38% of students reported that limited interactions were one of the least useful features of MyDispense [40]. Likewise, nearly three out of five students in another survey agreed that MyDispense has limited interactions [42], with some students noting that “*there were some limitations in discussing with patients*” [41].

### 3.5. Quality Appraisal

The MMAT critical appraisal tool was used to appraise studies across five categories. As can be seen in Table 3, only three studies achieved a “Yes” (Y) rating in all categories [39,43,47]. Mixed-methods studies dominate the list, and most are of moderate to high quality. Descriptive studies are generally well-rated, though some have unclear reporting. Quantitative RCTs are rare in this sample [24,40], attributed to the fact that randomisation of educational interventions for students such as MyDispense is inherently limited, and assessors or participants cannot be blinded prior to the study (criterion 2.4). Clarity of reporting is a common issue, as seen in the number of “can’t tell” (CT) ratings.

## 4. Discussion

This review identified facilitators and barriers to MyDispense use. Facilitators were categorised into four themes: (1) Develops Competency, (2) Accessibility, (3) Engaging Learning Experience and (4) Safe Learning Environment and barriers were encompassed by three themes: (1) Learning Curve, (2) IT issues, and (3) Limited Realism and Applications. Quality appraisal demonstrated that all included studies were generally of moderate to high quality.

This review explored MyDispense across a range of areas, e.g., pharmacotherapy and pharmacy law courses [26,34,35,36,37,38,39,40,41,42,43,44,45,46,47]. One facilitator which emerged was that MyDispense developed the required competencies for practice. Previous studies also identified that CBS can support competency and practical skill development in pharmacy students [15,28,35]. Pharmacy simulations act as low-demand alternatives to OSCEs, as they facilitate knowledge acquisition (“Knows How”) and knowledge applications (“Shows How”) in realistic scenarios, aligning with Millers educational framework [48,49]. Students can struggle to apply counselling skills in real-life scenarios when not provided with opportunities to practice in a high-fidelity environments [50]; however, the use of MyDispense ^TM^ can possibly overcome these issues to improve overall confidence in practice [51].

MyDispense provides an engaging, realistic learning experience with immediate feedback [26,41,43,44]. MyDispense is more engaging for students relative to traditional teaching methods [39]. In a survey exploring the global views of both students and educators on CBS usage in six World Health Organisation (WHO) regions, students were particularly positive about the engagement factor, with (72.4%, *n* = 177) finding CBS enjoyable and (77.6%, *n* = 190) agreeing that it was engaging [52]. Evidence also suggests that active learning methods can increase student engagement with lecture materials and improve performance in assessments [12]. Prompt feedback, which is a MyDispense feature, does not appear to improve student assessment performance, relative to traditional delayed feedback [53]. However, receiving such feedback in a timely manner can enhance student self-learning and metacognition, thereby promoting productive failure [26,54].

MyDispense was commonly employed during the COVID-19 pandemic, as educators explored innovative methods to substitute for traditional face-to-face teaching [41,44,47]. Virtual patients enable educators to provide a flexible, accessible, remote learning environment for students [28]. However, pharmacy students can feel socially isolated when online pedagogy is used and usually preference in-person learning, suggesting a balance needs to be struck by pharmacy educators and a blended learning approach should be employed when implementing MyDispense to meet student needs [55].

MyDispense provides a safe learning environment for students where they can make mistakes and repeat exercises without facing real-world repercussions [26,36,39,41,43,44]. This may be a useful feature for pharmacy students, who tend to be self-orientated perfectionists, as it provides them with ample opportunity to repeat exercises and correct mistakes [51,56]. This theme echoes the findings of a past review, concluding that high-fidelity simulations must provide a controlled environment to allow learners to focus on clinical skills without distraction whilst also having the opportunity for repetition to learn from mistakes to ensure an effective learning experience [57].

The initial learning curve of the simulation and IT issues were two barrier themes identified in this review. Initial difficulties appear to be common for other simulations used in pharmacy education [58]. Platform learning curves may be associated with inadequate digital literacy, as research underlined that enhanced digital competencies, improve student adaptability and assessment performance in blended learning environments [59]. Internet connection issues were most commonly reported by Vietnamese and Filipino students, suggesting infrastructural barriers to MyDispense [43,44]. This is supported by a recent survey reporting that only half of educators in the Western Pacific Region (WPRO) agree that their institution provides adequate technical support [52]. This indicates students in such regions may have limited technical support. Minor technical issues, however, appear to be universal to simulations used in pharmacy education [58,60]. Four out of five pharmacy students consider ease of use and bug-free experiences as essential features for simulations, emphasising how technical issues can serve as prominent barriers [27]. Institutions should employ technicians for platform troubleshooting and provide additional user guides, tailored for context and culture, to students to overcome such initial learning curve barriers; however, the establishment of such infrastructure can be costly and demanding for educators [52].

MyDispense only simulates community practice and limited aspects of hospital practice, e.g., discharge prescriptions, which is a barrier for student engagement and educational applications [26]. Other simulations, e.g., SimPharm^TM^ can simulate hospital pharmacies and can facilitate interprofessional learning (IPL) activities [9,20,61]. A previous review on CBS used in pharmacy education found that interaction elements of multiple simulations do have limited realism capabilities [25]. Despite this, MyDispense was designed to simulate community pharmacies [26] and to support the teaching of communication skills; therefore, educators should make students aware of its intended uses in pharmacy education prior to implementation.

### 4.1. Limitations

A limitation of this review was that studies published prior to 2015 were not included; however, it is unlikely that these studies would add significantly our findings as MyDispense is a novel simulation that was developed by Monash University in 2011 [26]. This review exclusively included studies published in English. As a result, relevant studies published in other languages may have been excluded, potentially limiting the comprehensiveness of the findings and introducing language bias.

### 4.2. Future Implications

The findings of this review suggest while pharmacy students perceive facilitators to using MyDispense, various factors can act as barriers to its adoption. The UI requires further work to provide a more-learner friendly experience. Opportunities could be explored by stakeholders to adapt MyDispense to wider cultural contexts and ensure its sustainability as a platform, by expanding the language database for international learners. Further research is warranted to explore stakeholders’ views on the barriers and facilitators to implementing simulations, e.g., MyDispense into pharmacy curricula. Identifying such challenges is the first step to inform future educators on successful implementation strategies to promote technology-enriched, diverse learning experiences for pharmacy students.

## 5. Conclusions

This review identified the barriers and facilitators to MyDispense use as reported by students. Across the reviewed literature, facilitators included, the development of required competencies, accessibility, engaging learning experience and safe learning environment, whilst barriers comprised a steep learning curve, overcoming technology challenges and the limited authenticity of the interactions. MyDispense allows for mistakes without facing real-life consequences, which facilitates its use in pharmacy education. Suggested improvements for MyDispense identified from this review were highlighted, and further development of the software is encouraged to enhance student engagement in future pharmacy education. The outcome of this review provides an understanding to educators of key factors to consider from the students’ perspective when implementing MyDispense into pharmacy curricula globally and may be useful for stakeholders in education when considering the implementation and use of MyDispense in the future.

## Figures and Tables

**Table 1:** PICO definitions.

PICO	Definitions
Population (P)	Pharmacy Students
Intervention (I)	Any study that collects pharmacy students’ opinion, perception, satisfaction or attitudes on using MyDispense in a recognised pharmacy course
Comparison (C)	Any type of study, i.e., with/without a comparison group
Outcomes (O)	Pharmacy students’ perceptions on the barriers and facilitators to using MyDispense

**Table 2:** Characteristics of included studies. (listed chronologically, according to most recent year of publication).

Author (Year); Country	Description of Study Design	Study Participants	Study Outcomes	Method(s) of Data Collection	Identified Barrier(s)	Identified Facilitator(s)
Waghel et al. (2025) USA [34]	Prospective, mixed methods, comparative study Cross-sectional	Y1 PharmD students enrolled in a pharmacy skills lab course (*n* = 71)	To evaluate the correlation between previous pharmacy experience and performance on MyDispense E&O activities To evaluate students perceptions of MyDispense	Post-intervention questionnaire investigating prior pharmacy experience and MyDispense perceptions	Initial learning curve to use software IT incompatibilities	Provides high fidelity learning interactive environment Provides immediate feedback Easy to navigate
Phanudulkitti et al. (2024) Thailand [20]	Quasi-intervention study with two rounds Longitudinal	Y4 Pharmacy students enrolled in a Pharmacotherapeutic I course (*n* = 136)	To evaluate MyDispense impact on pharmacy students’ learning outcomes To evaluate students perceptions along with instructors views of MyDispense	A five part mixed methods survey Part three comprised of five closed-ended questions about MyDispense perceptions and one item for additional student feedback	Learning how to use software initially	Can practice dispensing skills at any time or place Provides feedback instantly at end of exercises
Al-Diery et al. (2024) Qatar [42]	Quantitative, prospective study Longitudinal	Y1 pharmacy students enrolled in a Professional Skills II course (*n* = 55)	To evaluate impact of MyDispense on students self-reported reaction, learning and accuracy in dispensing tasks	Pre-post intervention seven-point Likert scale surveys based on Kirkpatricks Model	Does not stimulate true patient-practitioner interactions	Offers immediate feedback Allows for practice in a safe virtual dispensing environment
Nguyen et al. (2023) Vietnam [43]	Mixed methods study with two phases Longitudinal	Y4 and Y5 pharmacy students enrolled at UMP Vietnam (*n* = 69) Pharmacists with at least one year clinical practice experience (*n* = 23)	To investigate learners’ perspectives on effectiveness of MyDispense in learning dispensing skills To investigate the suitability of MyDispense integration into clinical pharmacy curriculums in Vietnam	Online five-point Likert scale questionnaire (phase 1) Semi-structured interview (phase II)	Complicated learning process of software Inconsistent quality of product images	High degree of user interactivity Ability to self-learn by immediate feedback Diverse medication database
Rude et al. (2023) USA [35]	Quantitative, prospective study Longitudinal	Y1 PharmD students enrolled at NDSU and VCU (*n* = 142)	To assess the impact of a MyDispense simulation on students knowledge and confidence of OTC medications To assess overall student perceptions of the MyDispense activity	Pre-post activity survey with closed-ended demographic, confidence and knowledge-based questions with a five modified-scale perception questions in the post-survey.	May not be as effective as traditional learning methods	Effective way to learn new information Encourages active thinking
Tabulov et al. (2023) USA [36]	Quantitative, prospective study Longitudinal	Y1 PharmD students enrolled in a pharmaceutical skills 1 course (*n* = 64)	To describe a paediatric simulation on MyDispensecompleted by first year students To review student perceptions on confidence and knowledge learned after using MyDispense simulation	Pre-post online questionnaire with yes/no items and a five-point Likert scale	Initial learning curve with the software is timely which may impact student engagement with MyDispense	Low-stakes environment that allows students to make mistakes without harm More realistic than paper-based case learning
Slater et al. (2023) United Kingdom [47]	Mixed methods, prospective study Cross-sectional	Y2 MPharm students enrolled in a pharmacy law and ethics module (*n* = 147)	To evaluate the impact of MyDispense simulation on assessment performance To evaluate student perceptions of MyDispense	Online post-simulation 24 item survey consisting of closed-ended and Likert-scale questions aswell as open-ended questions.	Software layout could be improved Difficulties navigating software initially	Highly accessible and can practice dispensing skills from home Provides opportunity to repeat exercises
Faller et al. (2022) Philippines [44]	Mixed methods, retrospective study Cross-sectional	Filipino pharmacy students from four universites that implemented MyDispense into their pharmacy cirricula (*n* = 322)	To determine learners level of perception of MyDispense	Online three-part survey including demographics, five-point Likert scale and open-ended questions on student perceptions of the software	IT and web connectivity issues	High-fidelity learning environment without patient harm
Amirthalingam et al. (2022) Saudi Arabia [45]	Mixed-methods, prospective study Cross-sectional	Y4 pharmacy students enrolled in an Introductory Pharmacy Practice Experience 2 course (*n* = 69)	To compare pharmacy students performance on MyDispense vs in-person OSCEs To explore students perceptions of MyDispense	Post-simulation questionnaire with a five-point Likert scale and open-ended questions	Can be complicated to use Robotic in nature	Helps improve patient communication skills Enhances student confidence in patient care
Deneff et al. (2021) USA [37]	Two year, mixed methods, prospective study Cross-sectional	Y3 PharmD students enrolled in a pharmacy law and ethics course in 2017 (*n* = 38) and 2018 (*n* = 28)	To evaluate the utility of and student perceptions on the usefulness of MyDispense for pharmacy law instruction	A qualitative survey with a series of close-ended questions graded on a four- and five-point Likert Scale in 2017 and 2018 respectively and open ended questions;	Challenges learning software cases without a tutorial Some pharmacy law exercises may not be suitable for MyDispense	More engaging than traditional classroom teaching
Ambroziak et al. (2018) USA [38]	Prospective, quantitative, comparative study Longitudinal	Y1 PharmD students enrolled in a Pharmacy Practice Skills 1 course (*n* = 85)	To implement MyDispense cases into Pharmacy Practice Skills 1 To assess student perceptions of their learning using MyDispense	Pre simulation survey investigating prior pharmacy experience Post simulation survey investigating perceptions of MyDispense using open and closed ended questions	Learning how to navigate program	Effective tool to learn dispensing skills e.g. analysing prescriptions
Ferrone et al. (2017) USA [39]	Prospective, mixed methods study Cross-sectional	Y1 and Y3 PharmD students enrolled in UCSF, UConn, STLCOP (*n* =241)	To implement MyDispense simulation into US pharmacy curricula To assess student satisfaction of the MyDispense simulation	Post-simulation survey with a five-point Likert scale, demographic query on pharmacy experience and open-ended questions on perceptions	Can be difficult to learn at first May need to be adapted for different regions to be more realistic in nature	Straightforward to learn Affords opportunity to learn and make mistakes without harming patients More realistic than paper based cases
Shin et al. (2016) USA [40]	Comparative, quantitative study Longitudinal	Y2 PharmD students enrolled in a Therapeutics II course (*n* = 117)	To demonstrate feasibility of integrating MyDispense into a therapeutics course To measure students perceptions on MyDispense and its impact on their learning	Pre-case and post-case tests Three post-intervention quantitative surveys consisting of 10 to 17 items	Limited capacity to simulate interactions with prescribers and patients	Provides immediate feedback Can practice cases at any time or place Provides safe, low stakes practice environment
McDowell et al. (2016) Australia [26]	Retrospective, mixed methods study Cross-sectional	Y1 BPharm students enrolled in PAC1311 and PAC1322 modules at Monash University (*n* = 199)	To develop MyDispense for students to learn dispensing skills in a low-stakes environment To explore student perceptions of MyDispens as a learning tool	38 item survey with five point Likert-scale questions and open ended questions	User interface is not responsive Technical and server connectivity issues	Learning support that allows “safe” dispensing without patient harm Stimulating learning environment
Dameh (2015) UAE [46]	Prospective, mixed-methods study Cross-sectional	Y2 female pharmacy students enrolled at FCHS (*n* = 33)	To report pharmacy students’ experience after using MyDispense	Survey consisting of five point Likert-Scale and open ended questions on student perceptions Focus group discussion to allow students to elaborate perceptions	Technical issues cause student frustration	Highly accessible and user friendly to students Gives dispensing practice prior to working in real-life scenarios

PharmD: Doctor of Pharmacy; MPharm: Masters of Pharmacy; Y1: Year 1; Y2: Year 2; Y3: Year 3; Y4: Year 4; E&O: Errors and omissions; OSCE: Objective Structured Clinical Examination; UMP: University of Medicine & Pharmacy Ho Chi Minh City; NDSU: North Dakota State University; VCU: Virginia Commonwealth University; UCSF: University of California, San Francisco; UConn: University of Connecticut; STLCOP: St. Louis College of Pharmacy; FCHS: Fatima College of Health Sciences; PAC1311: Pharmacy, Health and Society I; PAC1322: Pharmacy, Health and Society II.

**Table 3:** Quality Appraisal according to Mixed Methods Quality Appraisal Tool (MMAT) criteria [1]. (listed chronologically, according to publication year).

Category of Study Designs	MMAT Quality Criteria	Waghel et al. (2025) [34]	Phanudulikitti et al. (2024) [20]	Al-Diery et al. (2024) [42]	Nguyen et al. (2023) [43]	Rude et al. (2023) [35]	Tabulov et al. (2023) [36]	Slater et al. (2023) [47]	Faller et. al. (2022) [44]	Amirthalingm et al. (2022) [45]	Deneff et al. (2021) [37]	Ambroziak et. al. (2018) [38]	Ferrone et al. (2017) [39]	Shin et al. (2016) [40]	McDowell et al. (2016) [26]	Dameh et al. (2016) [46]
Qualitative	*1.1*	Y	Y	N/A	Y	N/A	N/A	Y	Y	Y	Y	Y	Y	N/A	Y	Y
	*1.2*	Y	Y		Y			Y	Y	Y	Y	Y	Y		Y	Y
	*1.3*	Y	CT		Y			Y	Y	Y	Y	CT	Y		Y	CT
	*1.4*	Y	Y		Y			Y	Y	Y	CT	CT	Y		Y	N
	*1.5*	CT	Y		Y			Y	Y	Y	Y	CT	Y		Y	N
Quantitative Randomized Controlled Trials (RCTs)	*2.1*	N/A	Y	N/A	N/A	N/A	N/A	N/A	N/A	N/A	N/A	N/A	N/A	Y	N/A	N/A
	*2.2*		N											Y		
	*2.3*		Y											Y		
	*2.4*		N											N		
	*2.5*		Y											Y		
Quantitative non-randomized	*3.1*	N/A	N/A	Y	N/A	Y	Y	N/A	N/A	N/A	N/A	N/A	N/A	Y	N/A	N/A
	*3.2*			Y		Y	CT							Y		
	*3.3*			Y		Y	CT							Y		
	*3.4*			CT		CT	Y							Y		
	*3.5*			Y		Y	Y							Y		
Quantitative descriptive	*4.1*	Y	N/A	N/A	Y	N/A	N/A	Y	Y	Y	Y	Y	Y	N/A	Y	Y
	*4.2*	Y			Y			Y	Y	Y	Y	Y	Y		Y	Y
	*4.3*	Y			Y			Y	CT	Y	Y	CT	Y		Y	Y
	*4.4*	Y			Y			Y	Y	CT	CT	Y	Y		Y	CT
	*4.5*	Y			Y			Y	Y	Y	Y	CT	Y		Y	N
Mixed Methods	*5.1*	Y	Y	N/A	Y	N/A	N/A	Y	Y	Y	Y	Y	Y	N/A	Y	Y
	*5.2*	Y	Y		Y			Y	Y	Y	Y	Y	Y		Y	CT
	*5.3*	Y	Y		Y			Y	Y	Y	Y	CT	Y		Y	N
	*5.4*	Y	Y		Y			Y	Y	Y	Y	Y	Y		Y	CT
	*5.5*	Y	N		Y			Y	Y	Y	Y	CT	Y		Y	N

Y: Yes, N: No, CT: Cant Tell, N/A: Not-Applicable.

**Figure A1:**
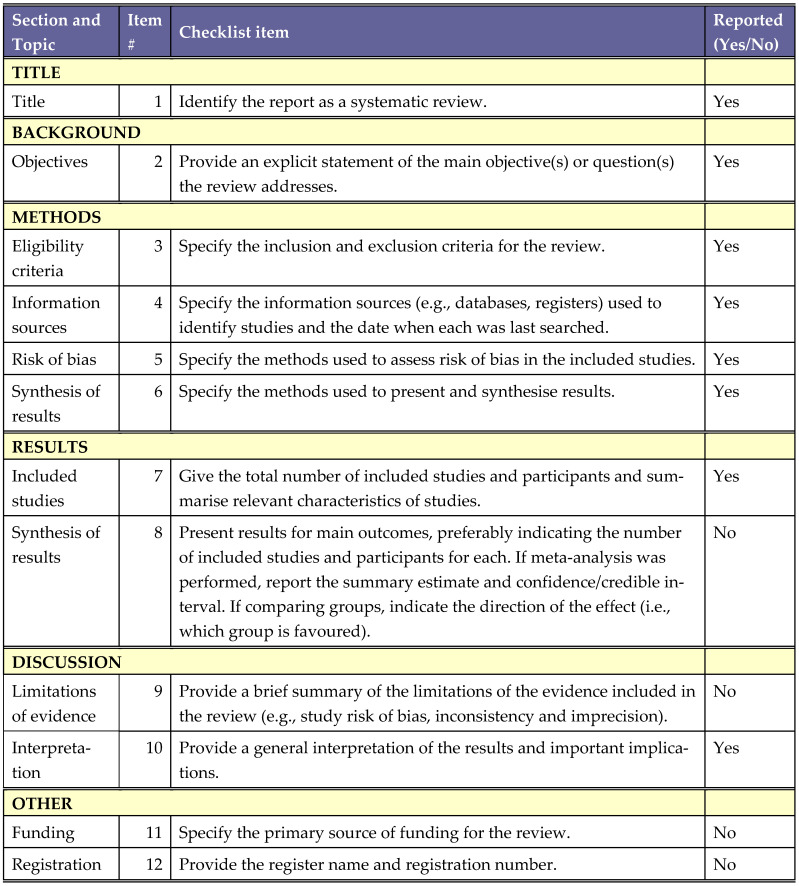
PRISMA 2020 Checklist for Abstract.

**Figure A2:**
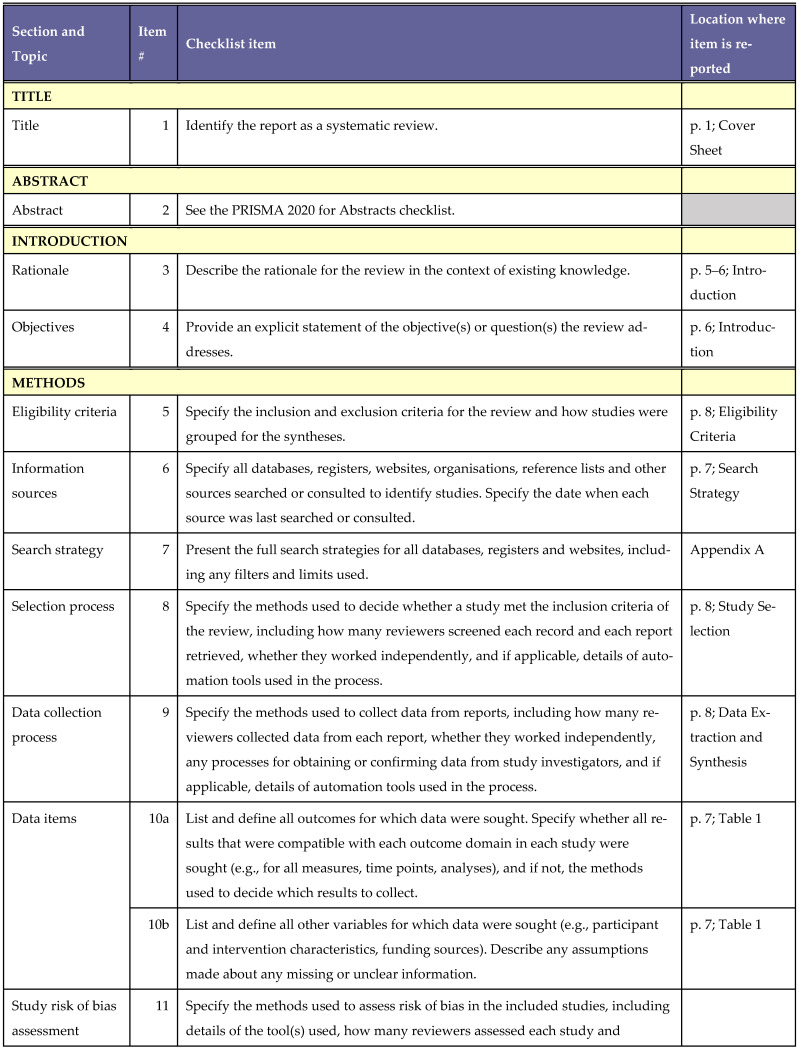

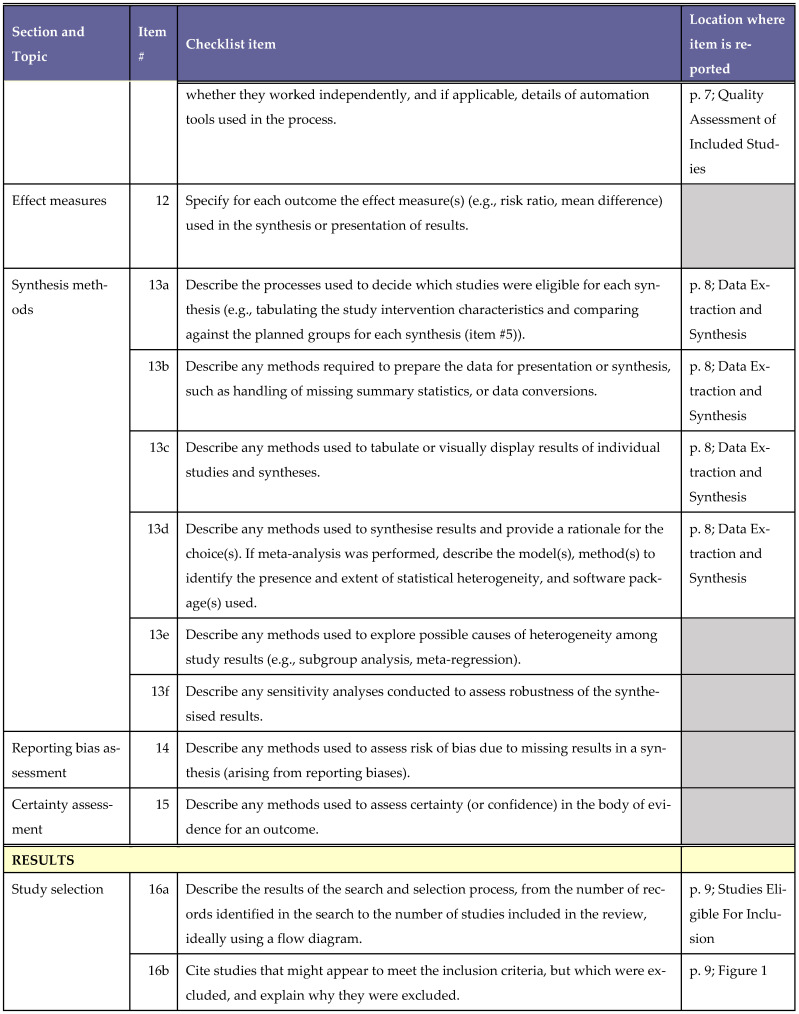

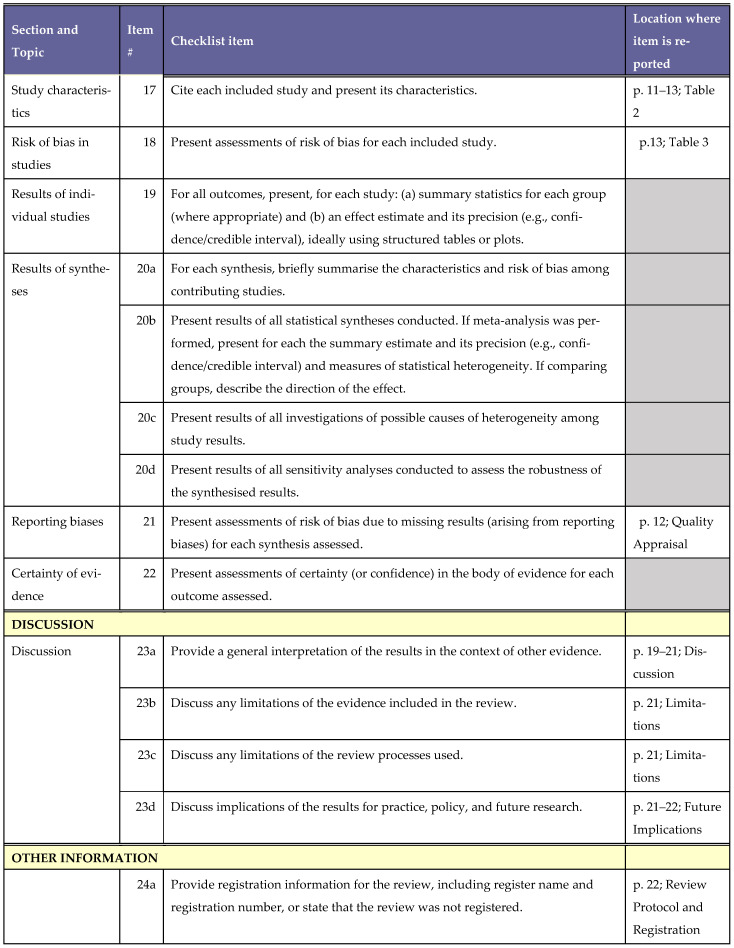

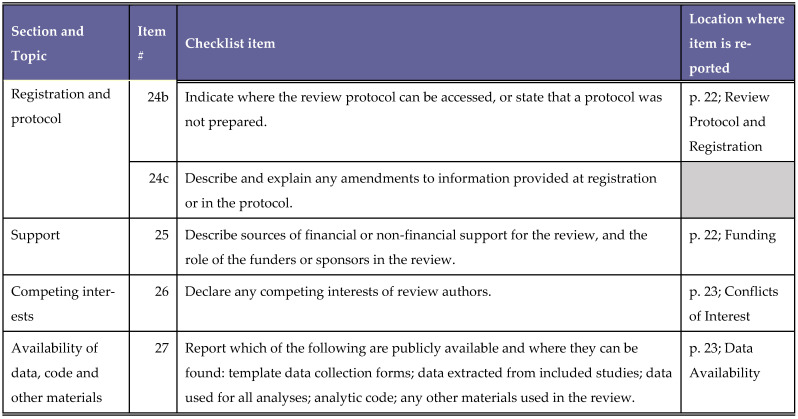
ISMA 2020 Checklist for Systematic Reviews.

## Data Availability

Not applicable.

## Appendix B. Search Strategy

A search on the PubMed, CINAHL and Embase databases were performed in January 2025 with four search strings (S1, S2, S3, S4) combined using the Boolean operator “AND” with the following limits set: year of publication 2015–2025. An example of the search and terms used for PubMed, CINAHL and Embase can be found, respectively, in Table A1, Table A2 and Table A3.

**Table A1:** PubMed Search Strategy.

Database	Date of Search	Search Strings	Terms Used	Results
PubMed	28 January 2025	S1	(perception[MeSH Terms]) OR (attitude[MeSH Terms])) OR (facilitator)) OR (enabler)) OR (barrier)) OR (obstacle)) OR (challenge)	4,224,261
		S2	(“MyDispense”) OR (computer simulation[MeSH Terms])) OR (patient simulations[MeSH Terms])) OR (educational technologies[MeSH Terms])) OR (“virtual patient simulator”[tiab:~3])) OR (“dispensing simulation”)	440,869
		S3	((students[MeSH Terms]) OR (pharmacy students[MeSH Terms]))	185,856
		S4	((pharmacy[MeSH Terms]) OR (pharmacy education[MeSH Terms]))	26,507
		S5	S1 AND S2 AND S3 AND S4	49

**Table A2:** CINAHL Search Strategy.

Database	Date of Search	Search Strings	Terms Used	Results
CINAHL	28 January 2025	S1	(MM “Attitude”) OR “beliefs” OR “views” OR “opinions” OR “barriers” OR “challenges” OR “obstacles” OR “facilitators” OR “enablers”	567,858
		S2	“Mydispense” OR “patient simulation” OR “virtual simulation” OR “computer simulation” OR “simulation” N2 (“patient” OR “virtual” OR “dispensing”)	27,672
		S3	(MH “Students”) OR (MH “Students, Pharmacy”)	22,056
		S4	(MH “Education, Pharmacy”) OR “pharmacy”	13,743
		S5	S1 AND S2 AND S3 AND S4	5

**Table A3:** Embase Search Strategy.

Database	Date of Search	Search Strings	Terms Used	Results
Embase	28 January 2025	S1	‘attitude’/de OR ‘attitude’ OR ‘beliefs’/de OR ‘beliefs’ OR ‘perception’/de OR ‘perception’ OR ‘challenge’/de OR ‘challenge’ OR ‘obstacles’/de OR ‘obstacles’ OR ‘barriers’/de OR ‘barriers’ OR ‘facilitator’/de OR ‘facilitator’ OR enablers	2,003,993
		S2	‘mydispense’ OR ‘computer simulation’/exp OR ‘computer simulation’ OR ‘patient simulation’/exp OR ‘patient simulation’ OR ((virtual OR patient OR dispensing) NEAR/2 simulation)	197,410
		S3	‘student’/exp OR ‘student’ OR ‘pharmacy student’/exp OR ‘pharmacy student’	617,087
		S4	‘pharmacy’/exp OR pharmacy OR ‘pharmacy education’/exp OR ‘pharmacy education’	1,299,905
		S5	S1 AND S2 AND S3 AND S4	117
